# Vancomycin-Induced Fever and Neutropenia in an Immunocompetent Patient With Complicated Community-Acquired Pneumonia

**DOI:** 10.7759/cureus.26630

**Published:** 2022-07-07

**Authors:** Hind H Alharbi, Ghaya I Al-Qurainees, Abdulqader Al-Hebshi

**Affiliations:** 1 Pediatrics, Prince Mohammed bin Abdulaziz Hospital, National Guard Health Affairs, Madina, SAU; 2 Pediatrics, King Saud Bin Abdulaziz University for Health Sciences, Riyadh, SAU; 3 Pediatric Infectious Diseases, Prince Mohammed bin Abdulaziz Hospital, National Guard Health Affairs, Madina, SAU; 4 Pediatric Hematology-Oncology, Ministry of National Guard Health Affairs, Madina, SAU

**Keywords:** skin rash, vancomycin, neutropenia, leukopenia, drug fever

## Abstract

Drug-induced fever can be caused by many medications through several mechanisms. One of the most common mechanisms is an immunologic reaction mediated by drug-induced antibodies.

Herein, we report the case of a rare adverse reaction with vancomycin. A six-year-old girl being treated for necrotizing pneumonia with vancomycin developed mild neutropenia, skin rash, and fever two weeks into her therapy. These resolved after stopping vancomycin, with noted reversal of neutropenia and leukopenia. Upon rechallenging the patient with vancomycin, she developed a fever in less than 24 h from the administration. Vancomycin-induced fever was made as a diagnosis of exclusion after all other possible causes were ruled out.

## Introduction

Drug-induced fever is defined as a fever occurring with the administration of a drug and disappearing after its discontinuation, and other causes for fever must be excluded after a careful physical examination and laboratory investigation [1]. Another definition is a febrile response to a drug without cutaneous manifestations [2]. Around 3% to 5% of cases with adverse drug reactions can present with fever [3-4]. Undiagnosed drug fever may prolong hospitalization with an extensive and expensive investigation. One study found that hospital stay was prolonged by a mean of 8.7 days per episode, generating an average of five blood cultures and 2.85 radiologic studies [4].

Vancomycin is a complex tricyclic glycopeptide antibiotic produced by Streptococcus orientalis;* *the new name of the organism is Amycolatopsis orientalis [5]. It is a bactericidal drug that inhibits bacterial cell wall biosynthesis [5]. It is generally used to treat severe infections caused by Gram-positive bacteria, particularly those caused by methicillin-resistant Staphylococci, and for patients with severe penicillin allergies [5]. Drug-induced fever from vancomycin is uncommon [6-7]. In some instances, fever has occurred alongside vancomycin-elicited neutropenia [8]. The hematologic manifestations of vancomycin-related reactions include leukocytosis, eosinophilia, neutropenia, and immune thrombocytopenia.

## Case presentation

**Figure 1:**
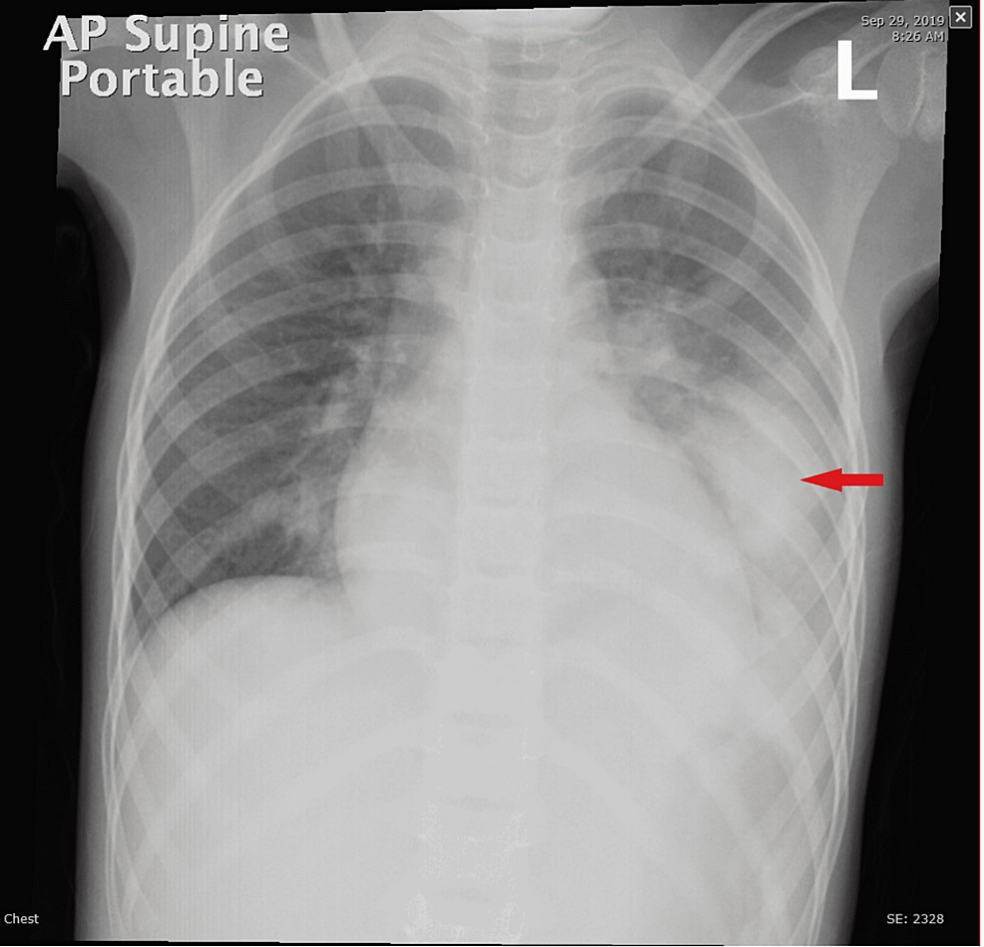
Chest X-ray Anteroposterior supine portable chest X-ray shows left-sided pneumonia (red arrow)

We report the case of complicated community-acquired pneumonia (CAP) in a previously healthy six-year-old girl. She presented with a four-day history of high-grade intermittent fever, nonproductive cough, and vomiting. Vital signs revealed a temperature of 38.5°C, blood pressure of 101/61 mmHg, respiratory rate of 35 breaths/min, and oxygen saturation of 96% on room air. Chest examination revealed crackles at the left lung base with bronchial breathing. Her chest X-ray (CXR) was consistent with left-sided pneumonia (Figure 1) while initial labs revealed leukocytosis. She also had elevations in C-reactive protein (CRP) and erythrocyte sedimentation rate (ESR) (Table 1).

**Table 1:** Laboratory investigations WBC, white blood cell count; Hb, hemoglobin; CRP, C-reactive protein; ESR, erythrocyte sedimentation rate

Test	Result	Reference range
WBC count	27 × 10^9 ^cells/L	(5-12 × 10^9^ cells/L)
Neutrophils	85.40%	(28%-60%)
Lymphocytes	13.30%	(28%-60%)
Eosinophils	0.76%	(1%-6%)
Monocytes	12.50%	(3%-9%)
basophils	0.05%	(0.5%-1%)
Hgb	12.4 g/dL	(12 to 16 g/dL)
Platelet	563x10^9^ cell/L	(150 – 450 × 10^9^ cells/L)
CRP	312.2 mg/L	(0.00–5.00 mg/L)
ESR	73 mm/h	(1–20 mm/h)

She was admitted to the general pediatric ward as a case of community-acquired pneumonia (CAP), and cefuroxime and azithromycin were begun. However, 72 h after admission, the patient still had a spiking fever with no clinical deterioration. Repeat CXR showed no interval changes compared to the first CXR, and the irregular patchy consolidation in the left lower lung zone and left-sided mild pleural effusion were seen again. Chest ultrasound showed evidence of minimal left-sided pleural effusion. The antibiotics were upgraded to ceftriaxone and vancomycin, while azithromycin was continued.

During vancomycin infusion, the patient developed Redman syndrome. Anaphylaxis was ruled out, and the rate of vancomycin infusion was decreased. The trough level had a high of 28 mcg/L, and thus, two doses were omitted before the dose was modified. The trough level of vancomycin was maintained at the therapeutic level between 15 and 20 mcg/ml. Ceftriaxone was changed to meropenem 48 h later since the patient continued to be febrile.

The patient showed clinical and laboratory improvement at 48 h on the last regimen; she became afebrile, WBC decreased to 17 × 10^9^ cells/L, and CRP decreased to 211.9 mg/L.

**Figure 2:**
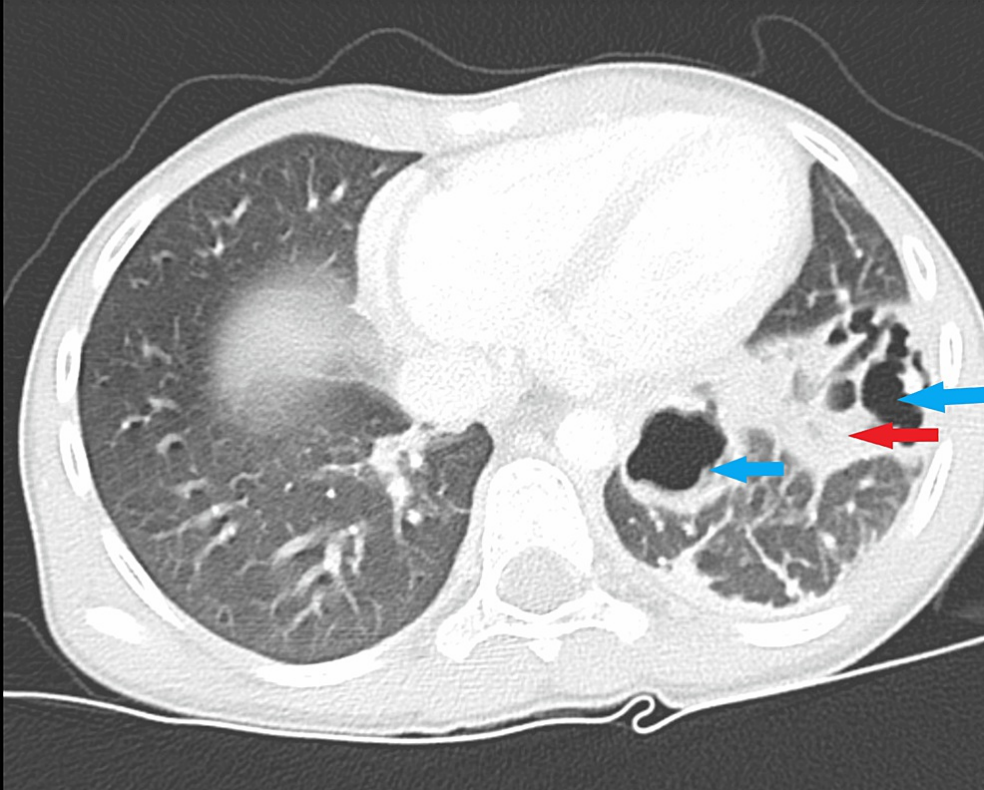
Computed tomography of the chest with contrast Anterior segment of left lower lung lobe consolidation (red arrow) with central necrosis and formation of multilocular cystic cavitation(blue arrow).

On the thirteenth day post-admission, the plan was to discharge the patient to continue her antibiotics at home, due to the clinical and laboratory improvement and decrease in CRP to 83.8 mg/L. However, oral medication was not tolerated, and thus, she was kept as an inpatient under the same regimen. On day 16, her fever spiked again, ranging from 38.6 to 39.5°C, with episodes three times a day that were responsive to antipyretics. There was no history of increasing cough, chest pain, abdominal pain, vomiting, diarrhea, or symptoms suggestive of urinary tract infection or peripheral line infection. Her WBC was 3.6 × 10^9^ cells/L, hemoglobin (Hg) was 120 g/L (normal: 125-155 g/L), platelet count was 450 × 10^9^ cells/L (normal: 150-450 × 10^9^ cells/L), ESR was 72 mm/h, and CRP was 16.3 mg/L. CXR showed patchy air space infiltration and suspicious cystic changes in the left lower lung zone. Chest computed tomography revealed anterior segment left lower lung lobe consolidation with central necrosis and formation of multilocular cystic cavitations, indicative of necrotizing pneumonia (Figure 2).

The patient continued to be febrile under the vancomycin and meropenem regimen despite negative blood and urine cultures and CXR suggestive of necrotizing pneumonia again. She reportedly felt well, with fever as her only complaint. CBC showed leukopenia, neutropenia, and lymphopenia (Table 2). The multiplex respiratory PCR test was negative.

**Table 2:** Laboratory investigations. WBC, white blood cell; ANC, absolute neutrophil count; ALC, absolute lymphocyte count

Test	Result	Reference range
WBC	3.2 × 10^9^/L	(5-12 × 10^9^ cells/L)
ANC	1.05 × 10^9^/L	(1.40–7.70 × 10^9^/L)
ALC	0.078 × 10^9^/L	(1.3–7.2 × 10^9^/L).

**Figure 3:**
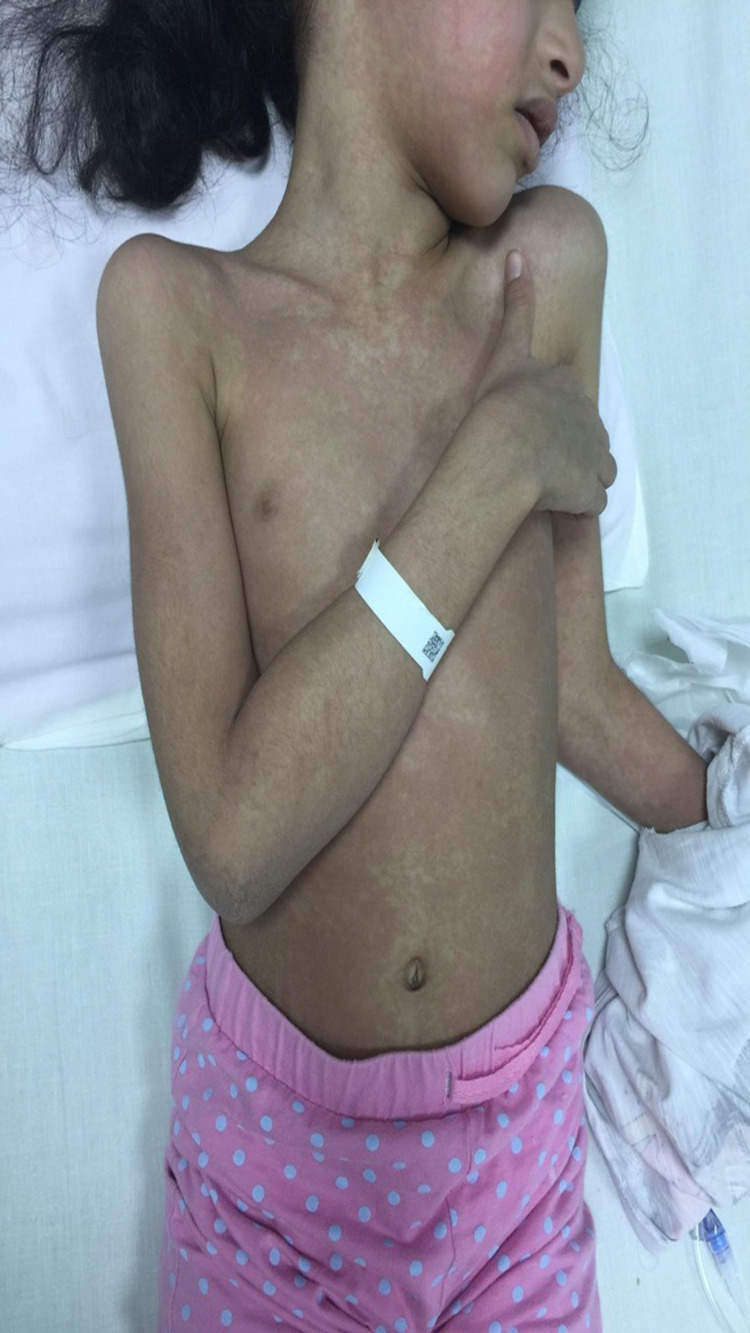
Skin rash A maculopapular rash over the trunk with distinctive slapped cheek appearance and circumoral pallor

On day 21, she developed a blanching, pruritic maculopapular rash initially localized to her trunk, which then became generalized, involving her entire body, with a distinctive “slapped cheek” appearance and circumoral pallor and desquamation of the feet (Figure 3). Complete physical examination revealed bilateral cervical lymph nodes, which were mobile, non-tender, and measuring 1 × 1 cm at the largest (right anterior area). There was no noted conjunctivitis. The rest of the chest examination was unremarkable, and CBC still showed pancytopenia with the same result. Since the patient still had spiking fever with the same rash and pancytopenia, she was worked up for other causes of prolonged fever; including tuberculosis, dengue, Brucella, Epstein-Barr virus, cytomegalovirus, parvovirus B19, and mycoplasma, which all were negative. Clinically, the patient did not meet the Kawasaki criteria, and her cardiac echocardiogram was normal. Immunodeficiency was also ruled out based on normal immunoglobulin levels and flow cytometry.

At this point, the cause of fever and neutropenia was most likely thought to be drug-induced. Meropenem was initially stopped (after 17 days) because we suspected it to be the most probable offending drug. However, the patient’s spiking fever persisted for the following three days, so vancomycin was stopped after 21 days of therapy. Afterward, the fever and neutropenia resolved within 72 h of discontinuing vancomycin, and repeated CBC revealed normalized results. We rechallenged the patient with one dose of vancomycin, and then the high-grade fever (39.4°C) quickly recurred within 3 h of administration, thus confirming our diagnosis of drug-induced neutropenia.

## Discussion

Upon reviewing the current literature, drug-induced fever has no specific type of pattern nor is it associated with a specific rash pattern. In the present case, the drug-induced fever was associated with rigors and a maculopapular rash involving her entire body, with a “slapped cheek” appearance on her face. Neutropenia is a rare adverse effect related to the prolonged use of vancomycin, occurring around seven to 20 days after initiation of treatment [9]. Our patient had a WBC count as low as 2 × 10^9^/L, with an absolute neutrophil count (ANC) reaching 1.08 × 10^9^/L. Both fever and neutropenia occurred in our patient two weeks after starting vancomycin.

The mechanism for vancomycin-induced neutropenia and fever is unclear. Multiple theories suggest an immunological basis, involving an immunoglobulin G (IgG) or IgM immune-mediated hypersensitivity reaction. This theory was supported by anti-granulocyte antibody positivity in the serum of a patient with vancomycin-induced neutropenia previously described by Schwartz [10]. It is postulated that the drug or another metabolite first binds to the host protein to form a drug-protein complex that acts as an antigen. Antibodies are then formed against that antigen, and the antigen-antibody immune complex then mediates complement activation, leading to the destruction of neutrophils [10]. This leads to neutropenia, and thus, lymphocytes are sensitized and pyrogenic substances are released, clinically manifesting as fever [11].

The administered drug can also be responsible for fever due to its composition or method of administration. Vancomycin stored for a period of time can have certain impurities, one of which is a powerful pyrogen (Mississippi mud) [12]. Modifications in the manufacturing process have reduced the occurrence of drug fever, but it has not been completely eliminated [13].

The most common mechanism of drug-induced fever is probably an immunologic reaction mediated by drug-induced antibodies. The fever usually occurs seven to 10 days of treatment, with no specific pattern of fever, and this usually resolves within 48 h of drug discontinuation. Failure to diagnose drug-induced fever can lead to inappropriate and potentially harmful diagnostic and therapeutic interventions. Thus, it is necessary to discontinue all potentially causative drugs. Performing a rechallenge with the offending agent will usually cause a recurrence of fever within a few hours, thus confirming the diagnosis [14].

## Conclusions

Vancomycin-induced drug fever with neutropenia was diagnosed by exclusion. We ruled out other alternative or coincidental diagnoses (i.e., foreign body), insufficient antibiotic coverage, the development of sequelae (e.g., lung abscess or empyema), and underlying immunodeficiency as causes of treatment failure. Furthermore, infection, connective tissue disorders, and cancer were all ruled out as possibilities. Vancomycin-induced drug fever required a high index of suspension to be diagnosed. Missing the diagnosis may lead to unnecessary work-up and medication or prolonged hospitalization.
